# An exploratory assessment of the management of pediatric traumatic brain injury in three centers in Africa

**DOI:** 10.3389/fped.2022.936150

**Published:** 2022-08-17

**Authors:** Madiha Raees, Shubhada Hooli, Amélie O. von Saint André-von Arnim, Tsegazeab Laeke, Easmon Otupiri, Anthony Fabio, Kristina E. Rudd, Rashmi Kumar, Patrick T. Wilson, Abenezer Tirsit Aklilu, Lisine Tuyisenge, Chunyan Wang, Robert C. Tasker, Derek C. Angus, Patrick M. Kochanek, Ericka L. Fink, Tigist Bacha

**Affiliations:** ^1^Division of Critical Care Medicine, Department of Anesthesia and Critical Care Medicine, The Children's Hospital of Philadelphia, Philadelphia, PA, United States; ^2^Department of Critical Care Medicine, University of Pittsburgh Medical Center (UPMC) Children's Hospital of Pittsburgh, Pittsburgh, PA, United States; ^3^Division of Pediatric Emergency Medicine, Department of Pediatrics, Baylor College of Medicine and Texas Children's Hospital, Houston, TX, United States; ^4^Division of Pediatric Critical Care, Department of Pediatrics, University of Washington and Seattle Children's Hospital, Seattle, WA, United States; ^5^Department of Global Health, University of Washington, Seattle, WA, United States; ^6^Division of Neurosurgery, Department of Surgery, College of Health Science, Addis Ababa University, Addis Ababa, Ethiopia; ^7^Department of Clinical Medicine, Faculty of Medicine, University of Bergen, Bergen, Norway; ^8^National Institute for Health Care and Research (NIHR) Global Health Research Group on Neurotrauma, University of Cambridge, Cambridge, United Kingdom; ^9^School of Public Health, Kwame Nkrumah University of Science and Technology, Kumasi, Ghana; ^10^Epidemiology Data Center, University of Pittsburgh, Pittsburgh, PA, United States; ^11^Department of Critical Care Medicine, University of Pittsburgh, Pittsburgh, PA, United States; ^12^Clinical Research, Investigation, and Systems Modeling of Acute Illness Center (CRISMA), University of Pittsburgh, Pittsburgh, PA, United States; ^13^Department of Paediatrics and Child Health, University of Nairobi, Nairobi, Kenya; ^14^Department of Pediatrics, Columbia University Medical Center, New York, NY, United States; ^15^Department of Paediatrics, University Teaching Hospital of Kigali, Kigali, Rwanda; ^16^Department of Epidemiology, Graduate School of Public Health, University of Pittsburgh, Pittsburgh, PA, United States; ^17^Department of Anesthesiology, Critical Care and Pain Medicine, Boston Children's Hospital, Boston, MA, United States; ^18^Safar Center for Resuscitation Research, University of Pittsburgh, Pittsburgh, PA, United States; ^19^Department of Pediatrics and Child Health, St. Paul Millennium Medical College, Addis Ababa, Ethiopia

**Keywords:** Africa South of the Sahara, global health, pediatrics, critical care, brain injuries, traumatic

## Abstract

**Purpose:**

Traumatic brain injury (TBI) is a leading cause of morbidity and mortality in low- and middle-income countries (LMICs). Hospital care practices of pediatric TBI patients in LMICs are unknown. Our objective was to report on hospital management and outcomes of children with TBI in three centers in LMICs.

**Methods:**

We completed a secondary analysis of a prospective observational study in children (<18 years) over a 4-week period. Outcome was determined by Pediatric Cerebral Performance Category (PCPC) score; an unfavorable score was defined as PCPC > 2 or an increase of two points from baseline. Data were compared using Chi-square and Wilcoxon rank sum tests.

**Results:**

Fifty-six children presented with TBI (age 0–17 y), most commonly due to falls (43%, *n* = 24). Emergency department Glasgow Coma Scale scores were ≤ 8 in 21% (*n* = 12). Head computed tomography was performed in 79% (*n* = 44) of patients. Forty (71%) children were admitted to the hospital, 25 (63%) of whom were treated for suspected intracranial hypertension. Intracranial pressure monitoring was unavailable. Five (9%, *n* = 5) children died and 10 (28%, *n* = 36) inpatient survivors had a newly diagnosed unfavorable outcome on discharge.

**Conclusion:**

Inpatient management and monitoring capability of pediatric TBI patients in 3 LMIC-based tertiary hospitals was varied. Results support the need for prospective studies to inform development of evidence-based TBI management guidelines tailored to the unique needs and resources in LMICs.

## Introduction

Despite global progress in reducing deaths in children, injury remains a significant contributor to mortality in children of all ages, with the majority of such events occurring in low- and middle-income countries (LMICs) (1–3). Traumatic brain injury (TBI) in particular is a leading cause of death and disability worldwide across all age groups (4–7). Patients with severe TBI in sub-Saharan Africa have nearly twice the mortality risk compared to those in high-income countries (HICs) (8).

Evidence- and consensus-based guidelines for the care of children with concussion and severe TBI published by HICs presume the availability of emergency transport, devices and technologies, expert medical and surgical personnel, rehabilitation services, and systems resources that are often unavailable in LMICs (9, 10). Recently published public policy recommendations highlight the need for all stakeholders to recognize and invest in expanded access to resources to improve outcomes for patients with head and spine injuries in LMICs; however, evidence to identify unmet needs to help prioritize resources for pediatric TBI care in LMICs is lacking (11).

The parent study for this work, the Prevalence of Acute critical Neurological disease in children: a Global Epidemiological Assessment-Developing Countries (PANGEA-DC), reported the epidemiology and outcomes of children presenting with TBI and infectious encephalopathy in four sub-Saharan African centers (12). This new secondary analysis aimed to provide a detailed description of the clinical management of children with TBI. The objective was to examine current clinical monitoring, testing, and therapeutics practices for children presenting with TBI.

## Methods

### Location

This was a secondary analysis of a prospective observational study conducted over a 4-week period (PANGEA-DC) (12). Three hospitals contributed data for TBI patients: Tikur Anbessa Hospital of Addis Ababa University (Addis Ababa, Ethiopia), Kenyatta National Hospital (Nairobi, Kenya), and University Teaching Hospital (Kigali, Rwanda). Sites started individual data collection shortly after local IRB approval was completed. Thus, Kenya and Rwanda sites collected data from October-November 2015 and Ethiopia from July-August 2015. The Ghana site from PANGEA-DC did not enroll any TBI patients during the study period and thus was not included in this secondary analysis. The study was approved by the Institutional Review Board of the University of Pittsburgh and at each study site.

All three study centers are public, tertiary referral centers with a university affiliation and locally available emergency medical transport systems. The highest level of training for pre-hospital care providers was basic life support, if any formal training had been completed at all. Availability of transport services varied substantially based on patient location, current traffic and equipment conditions, and ability to pay for services. Each center can provide non-invasive and invasive mechanical ventilation and perform routine laboratory studies and head computed tomography (CT) scans on site (Supplementary Table 1). All centers reported access to neurosurgical consultation but only the Ethiopia site reported 24-h access to a neurosurgeon. No site had existing local guidelines for management of pediatric TBI or a trauma system. No centers have invasive intracranial pressure (ICP) monitoring available for TBI management. Inpatient physical therapy (PT) was available in Rwanda and Kenya, and occupational therapy (OT) was available in Ethiopia and Kenya.

### Data collected

Children aged 0–18 years presenting to the emergency department (ED) with TBI were included in the analysis. Inclusion criteria consisted of a complaint of traumatic brain injury of any severity in a patient aged 18 years or younger. Exclusion criteria were history of bleeding disorder and pregnancy. Through manual paper chart review, sites collected patient demographics, injury and pre-hospital care, neurological status [e.g., Glasgow Coma Scale (GCS) score], laboratory and brain imaging studies performed in the first 24 h, highest level of care, organ supports and monitoring, therapeutics for clinically suspected intracranial hypertension (ICH), inpatient rehabilitation, and hospital discharge outcome. Clinicians were queried specifically about the following interventions used to treat presumed ICH: sedatives/analgesics, hyperosmolar therapy (hypertonic saline and/or mannitol), or decompressive craniectomy. Not all therapies were available for each patient. All inpatients treated for ICH had reported availability of sedatives and analgesics (e.g., opiates, barbiturates, etc.), along with the ability to perform decompressive craniectomy and administer mannitol. Hypertonic saline was only available to the patients enrolled from the Kenya site.

These data were entered by a combination of site principal investigators and research coordinators, depending on staff availability, and thus blinding was not consistently completed.

The primary objective was to report monitoring, testing, and therapeutic practices across the care continuum (pre-hospital, hospital, discharge). The primary outcome was patient neurological outcome, ranging from no disability to death, at hospital discharge using the Pediatric Cerebral Performance Category (PCPC) score (13). Favorable outcome was defined as a discharge PCPC score of 1 or 2, reflecting no or mild disability. An unfavorable outcome was defined as a discharge PCPC score of 3–6 or death, reflecting at least moderate disability, or a change of ≥2 from baseline (13).

### Study definitions

Physiologic parameter limitations were set based on published pediatric severe TBI guidelines (9). Hypotension was defined as systolic blood pressure <70 mmHg + age in years x2 up to 90 mmHg, hypothermia as temperature <36°C, hyperthermia as temperature **>**37.5°C, hypoxemia as oxygen saturation of <92%, bradycardia as heart rate <60 beats per minute, and tachycardia >120 beats per minute for children under 12, and >100 beats per minute for those age 12 and older. Severe TBI was defined as a presenting GCS score (obtained on arrival to the site ED) of 3–8, moderate as 9–13, and mild as 14–15. Suspected ICH was determined clinically by the treating team. Polytrauma was defined as patients having one non-neurological system injured and was determined by the treating team.

Descriptive statistics are presented as median with interquartile range (IQR) as data were non-parametric. When data for GCS score and discharge disposition were missing, patients were excluded, resulting in exclusion of two patients total from the initial cohort of 58 patients. In the remaining cases, missing data were not imputed; thus, when the total sample size for a variable was less than the number of enrolled patients (56), denominators are provided. Data were analyzed using Chi-square test and Wilcoxon rank sum test as appropriate. All *p-*values < 0.05 were considered statistically significant. Stata (College Station, TX, 2019) was used for statistical analysis.

## Results

### Patients and TBI presentation

**Figure 1:**
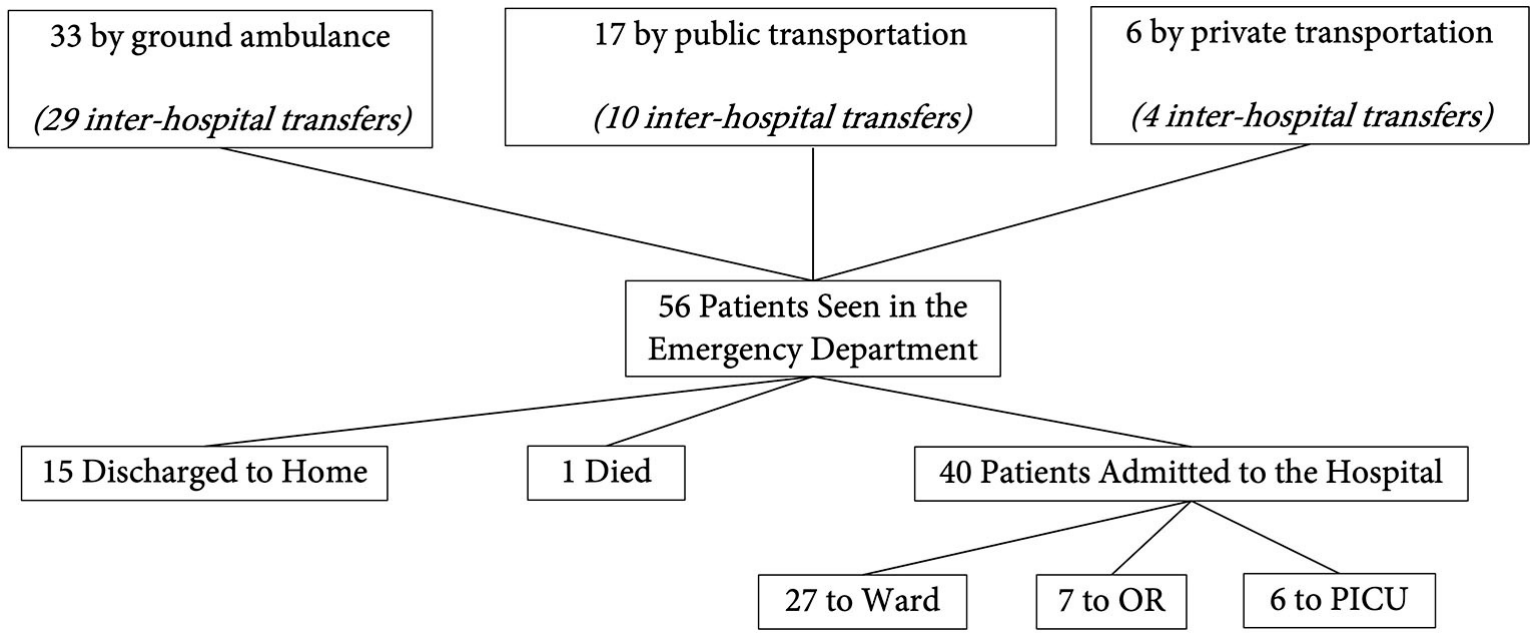
Patient flow diagram. This figure demonstrates the flow of our patients from the pre-hospital, emergency department, and in-hospital settings. OR, operating room; PICU, pediatric intensive care unit.

Fifty-six children presenting to the ED of one of our participating study centers with TBI were enrolled on admission and followed to hospital discharge. Thirty-seven (66%) patients were enrolled from Ethiopia, 7 (13%) from Kenya, and 12 (21%) from Rwanda. Table 1 contains demographic and injury details grouped by TBI severity. Most (96%, *n* = 54) patients were previously healthy. Twenty-seven percent (*n* = 15) were female. The most common causes of injury were falls (*n* = 24, 43%) followed by motorized vehicle accidents (*n* = 14, 25%). Twenty-six of 51 (53%) had polytrauma. Seventy-three percent (*n* = 41) of patients had a reported loss of consciousness at some point following injury. Figure 1 demonstrates patient flow from pre-hospital through hospital admission.

**Table 1:** Patient demographics and injury details by TBI severity.

**Median (IQR) or *n* (%)**	**Mild TBI**	**Moderate TBI**	**Severe TBI**
	***n =* 34**	***n =* 10**	***n =* 12**
Age, years	10 (5–13)	9 (6–12)	10.5 (3.5–13)
Female sex	8 (24)	3 (30)	4 (33)
Previously healthy	32 (94)	10 (100)	12 (100)
**TBI mechanism**			
Fall	13 (38)	5 (50)	6 (50)
Motor vehicle accident	7 (21)	4 (40)	3 (25)
Blunt object	8 (23)	1 (10)	1 (8)
Pedestrian struck	4 (12)	0 (0)	0 (0)
Other or Unknown	2 (6)	0 (0)	2 (17)
**Intention of injury**			
Accidental	28 (85)	9 (90)	10 (83)
Self-inflicted	0 (0)	1 (10)	0 (0)
Assault	4 (12)	0 (0)	1 (8)
Other/Unknown	1 (3)	0 (0)	1 (8)
Loss of Consciousness	19 (56)	10 (100)	12 (100)
Multiple trauma	13 (41), *n =* 32	7 (78), *n =* 9	6 (75), *n =* 8
Pre-PCPC Score 1–2	33 (100), *n =* 33	9 (100), *n =* 9	10 (83)
Post-PCPC Score 1–2	29 (88), *n =* 33	7 (78), *n =* 9	3 (25)
**ED disposition**			
Home	14 (41)	1 (10)	0 (0)
Inpatient ward	17 (50)	6 (60)	4 (33)
Operating room	3 (9)	3 (30)	1 (8.5)
PICU	0 (0)	0 (0)	6 (50)
Died	0 (0)	0 (0)	1 (8.5)

IQR, interquartile range; TBI, traumatic brain injury; GCS, Glasgow Coma Scale; PCPC, Pediatric Cerebral Performance Category; ED , emergency department; PICU, pediatric intensive care unit.

This table contains information on patient demographic and injury details separated by severity of TBI determined by admission GCS (mild TBI is defined as GCS 14–15, moderate as GCS 9–13, and severe as 3–8). Column header n represents number of patients in each group total, while individual cell n represents number of patients in that particular category. “Motorized vehicle” refers to a passenger struck in a motor vehicle or motorcycle accident while “pedestrian struck” is a pedestrian struck by a motorized or non-motorized vehicle. Variable sample sizes are due to missing data.

### Pre-hospital

Seventy-seven percent (*n* = 43) of patients were transferred to the study center from another medical facility. Of these patients, two thirds (67%, *n* = 29) were transported by ground ambulance with the remaining patients brought in by public (23%, *n* = 10) or private (10%, *n* = 4) transport. Basic life support trained personnel represented the highest level of care available for medical transportation for all patients. Median distance traveled to the referral center was 60 kilometers (IQR 10–147 km). Median transport time was 80 min (IQR 40–180 min).

### Emergency department

Physiological parameters were not consistently measured across patients with 31 patients (55%) having a complete set of vital signs recorded (blood pressure, heart rate, temperature, respiratory rate, and oxygen saturation). Heart rate was measured in 89% (*n* = 50), axillary temperature in 88% (*n* = 49), respiratory rate in 86% (*n* = 48), oxygen saturation in 80% (*n* = 45), and blood pressure in 60% (*n* = 33). For those with vital signs recorded, 15% were hypotensive for age (5/33), 8% were hypothermic (4/49), 8% were hyperthermic (4/49), and 13.5% were hypoxemic (6/45) (Table 2).

**Table 2:** Initial emergency department physiologic assessment and management by TBI severity.

**Median (IQR) or *n* (%)**	**Mild TBI**	**Moderate TBI**	**Severe TBI**
	***n =* 34**	***n =* 10**	***n =* 12**
Hypothermia	3 (10), *n =* 30	1 (10)	0 (0), *n =* 9
Hyperthermia	2 (7), *n =* 30	1 (10)	1 (11), *n =* 9
Hypotension	2 (11), *n =* 19	2 (29), *n =* 7	1 (14), *n =* 7
Hypoxia	6 (21), *n =* 28	4 (44), *n =* 9	5 (63), *n =* 8
Bradycardia	1 (3), *n =* 29	0 (0), *n =* 7	1 (11), *n =* 9
Tachycardia	2 (7), *n =* 29	3 (43), *n =* 7	3 (33), *n =* 9
**Initial respiratory support**			
None	0 (0)	7 (70)	2 (17)
Supplemental oxygen	1 (3)	3 (30)	9 (75)
Bag-valve mask	0 (0)	0 (0)	1 (8)
ED endotracheal intubation	0 (0)	0 (0)	1 (8)

IQR, interquartile range; TBI, traumatic brain injury; GCS, Glasgow Coma Scale; ED, emergency department; PICU, pediatric intensive care unit.

This table contains information on patient physiological parameter assessment and initial respiratory support provided in the emergency department separated by severity of TBI determined by admission Glasgow Coma Scale score. Hypotension was defined as systolic blood pressure <70 mmHg + age in years x2 up to 90 mmHg, hypothermia as temperature <36°C, hyperthermia as temperature **>** 37.5°C, hypoxemia as oxygen saturation of <92%, bradycardia as heart rate <60 beats per minute, and tachycardia >120 beats per minute for children under 12, and >100 beats per minute for those age 12 and older. Severe TBI was defined as a presenting GCS score of 3–8, moderate as 9–13, and mild as 14–15. Column header n represents number of patients in each group total, while individual cell n represents number of patients in that particular category. Variable sample sizes are due to lack of uniform vital sign measurement.

Forty children were admitted to the hospital, 15 children were discharged home, and one child died in the ED. The median ED GCS score of those children admitted to the ward was 15 (12–15, *n* = 27), to the pediatric intensive care unit (PICU) 6.5 (5–7, *n* = 6), and of those taken directly to the operating room (OR) 12 (10–15, *n* = 7). Three of 15 (20%) patients discharged from the ED had a presenting GCS <15, which may be due to the fact that serial GCS measurements were not collected. Two thirds (27/41) of males were admitted compared to 87% (13/15) of females, correlating with proportions of TBI severity in males (63% mild [*n* = 26], 17% moderate [*n* = 7], 20% severe [*n* = 8]) vs. females (53% mild [*n* = 8], 20% moderate [*n* = 3], 27% severe [*n* = 4]).

The majority (34/40, 85%) of patients who were admitted to the hospital had laboratory values analyzed on admission; the most common abnormality was anemia (74%, 25/40). No patients had electrolytes outside of sodium and glucose or markers of end organ function checked (e.g., creatinine, alanine aminotransferase, aspartate aminotransferase). All of the patients who were transferred to the OR directly from the ED had a white blood cell count, hemoglobin, and hematocrit measured. Blood sodium testing was performed in eight patients, five of whom had hyponatremia (sodium <135 mEq/L). Three patients had an arterial blood gas with lactate analyzed in the ED, one of which demonstrated an acidosis with an abnormally elevated lactate (>2 mmol/L). No other labs were obtained in the emergency department.

**Figure 2:**
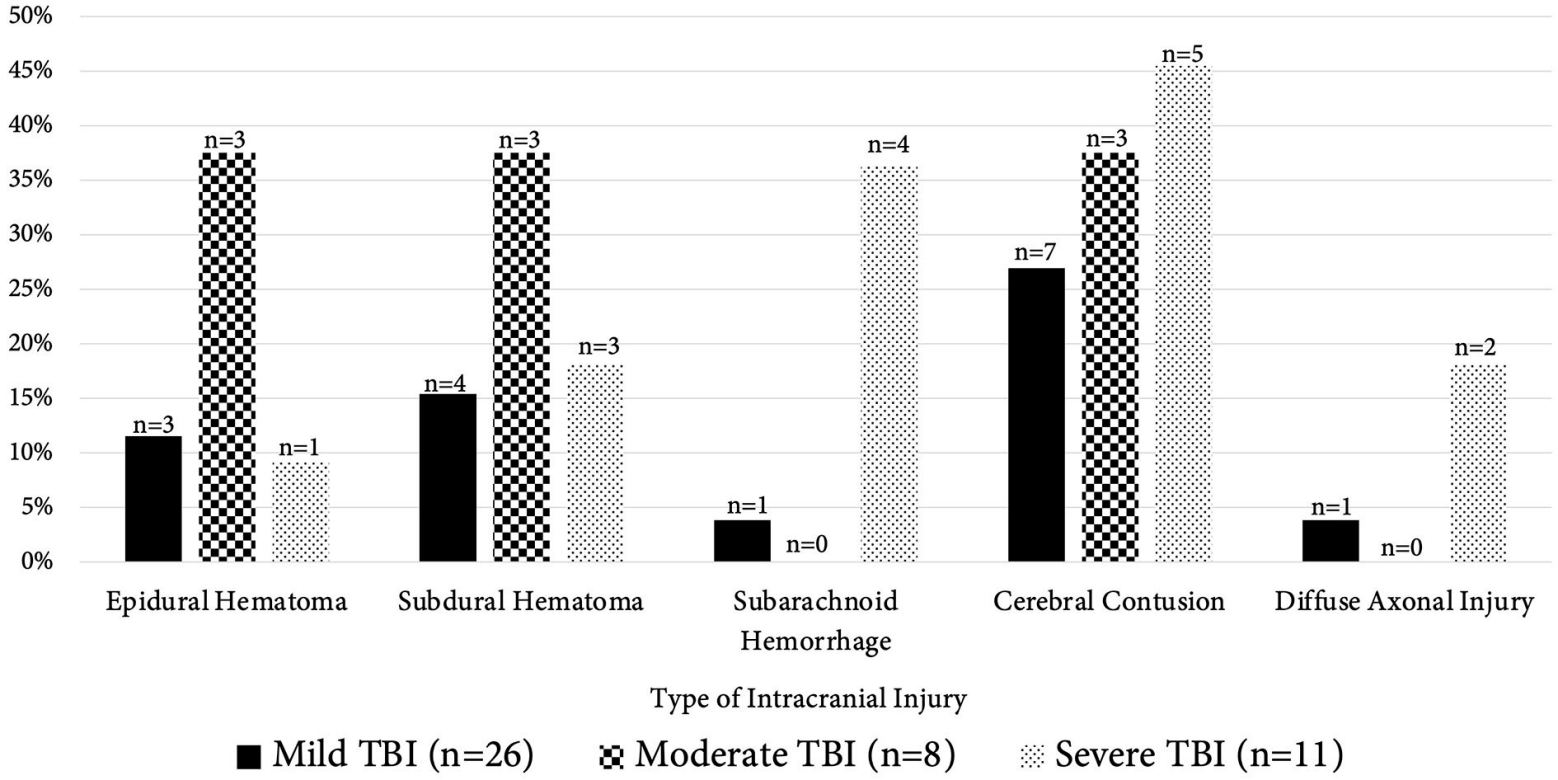
Abnormal computed tomography findings by TBI severity. This figure demonstrates the distribution of abnormal head computed tomography findings in patients between TBI severity groups (mild TBI [GCS score 14–15, black columns], moderate TBI [GCS 9–13, checkerboard columns], severe TBI [GCS 3–8, dotted columns]). The x-axis contains the various abnormal intracranial findings noted on imaging; the “*n*” at the top of the bar represents total number of patients that had that particular radiographic finding while total group n of patients with imaging in each TBI severity is given in the lower legend (mild TBI, *n* = 26; moderate TBI, *n* = 8; severe TBI, *n* = 11). The y-axis represents the percentage of patients that had those radiographic findings. TBI, traumatic brain injury.

A head CT was performed in 44 (76%) patients, 82% (36/44) of which had abnormal results, detailed in Figure 2. The most common abnormality was cerebral contusion (15/44, 34%) followed by subdural hematoma (9/44, 20%). Skull fractures and extra-calvarial injuries were not classified, nor was the presence of cerebral edema, midline shift, or herniation. Six patients had multiple abnormalities noted.

No vasopressors or inotropes were initiated in the ED. One patient required endotracheal intubation in the ED and was admitted to the PICU.

### Inpatient

As mentioned previously, 40 children (71%) were admitted to the hospital; 27 to the inpatient ward, 6 to the PICU, and 7 were taken directly to the OR from the ED. In the PICU, continuous pulse oximetry was used in all six patients, while 5/6 were monitored with a continuous cardiovascular monitor. One had a central venous catheter and two had arterial catheters placed. On the ward, continuous pulse oximetry was more common (19/27, 70%) than cardiovascular monitoring (2/27, 7%). Table 3 details monitoring and interventions carried out in patients classified by their initial touchdown location after ED admission.

**Table 3:** Inpatient monitoring and interventions separated by highest level of care.

**Median (IQR) and *n* (%)**	**Ward**	**Operating room**	**PICU**
	***n =* 27**	***n =* 7**	***n =* 6**
Continuous pulse oximetry	19 (70)	5 (71)	6 (100)
Continuous cardiac rhythm monitoring	2 (8)	3 (43)	5 (83)
Central venous catheter	0 (0)	0 (0)	1 (20)
Arterial catheter	0 (0)	0 (0)	2 (40)
Mechanical ventilation	0 (0)	0 (0)	5 (83)
**ICH directed therapy**			
Any ICH directed therapy	10 (37)	6 (86)	5 (83)
Mannitol	2 (11), *n =* 19	1 (33), *n =* 3	4 (67)
Hypertonic saline	0 (0), *n =* 4	0 (0), *n =* 1	1 (33), *n =* 3
Decompressive craniectomy	4 (15)	3 (43)	3 (50)
Intravenous fluids	9 (33)	2 (29)	5 (83)
Tube feeds	3 (23), *n =* 12	1 (25), *n =* 4	3 (60), *n =* 5
Inpatient physical therapy	6 (43), *n =* 13	1 (33), *n =* 3	2 (67), *n =* 3
Inpatient occupational therapy	0 (0), *n =* 4	*n =* 0	1 (33), *n =* 3

IQR, interquartile range; ICH, intracranial hypertension; ED, emergency department; PICU, pediatric intensive care unit; PCPC, pediatric cerebral performance category.

This table contains the presence of invasive and non-invasive monitoring devices, therapeutic interventions, and outcomes for all hospitalized patients. Patients are separated in groups determined by the location of their first touchdown clinical space in the hospital. Column header n represents number of patients in each group total, while individual cell n represents number of patients that had the particular therapy available to them during their hospitalization, resulting in variable sample size.

No inotropes or vasopressors were used for any hospitalized patient (*n* = 40). Five patients were mechanically ventilated (4/5 were intubated after admission from ED) (Table 3); all had a presenting GCS ≤ 8. Median duration of mechanical ventilation was 7 days (IQR 5–11). No additional GCS scores were obtained. A standard EEG was performed for one patient admitted to the ward who experienced clinical seizures on day 1 of hospitalization. No patient had continuous EEG, brain ultrasound, or brain magnetic resonance imaging performed.

**Figure 3:**
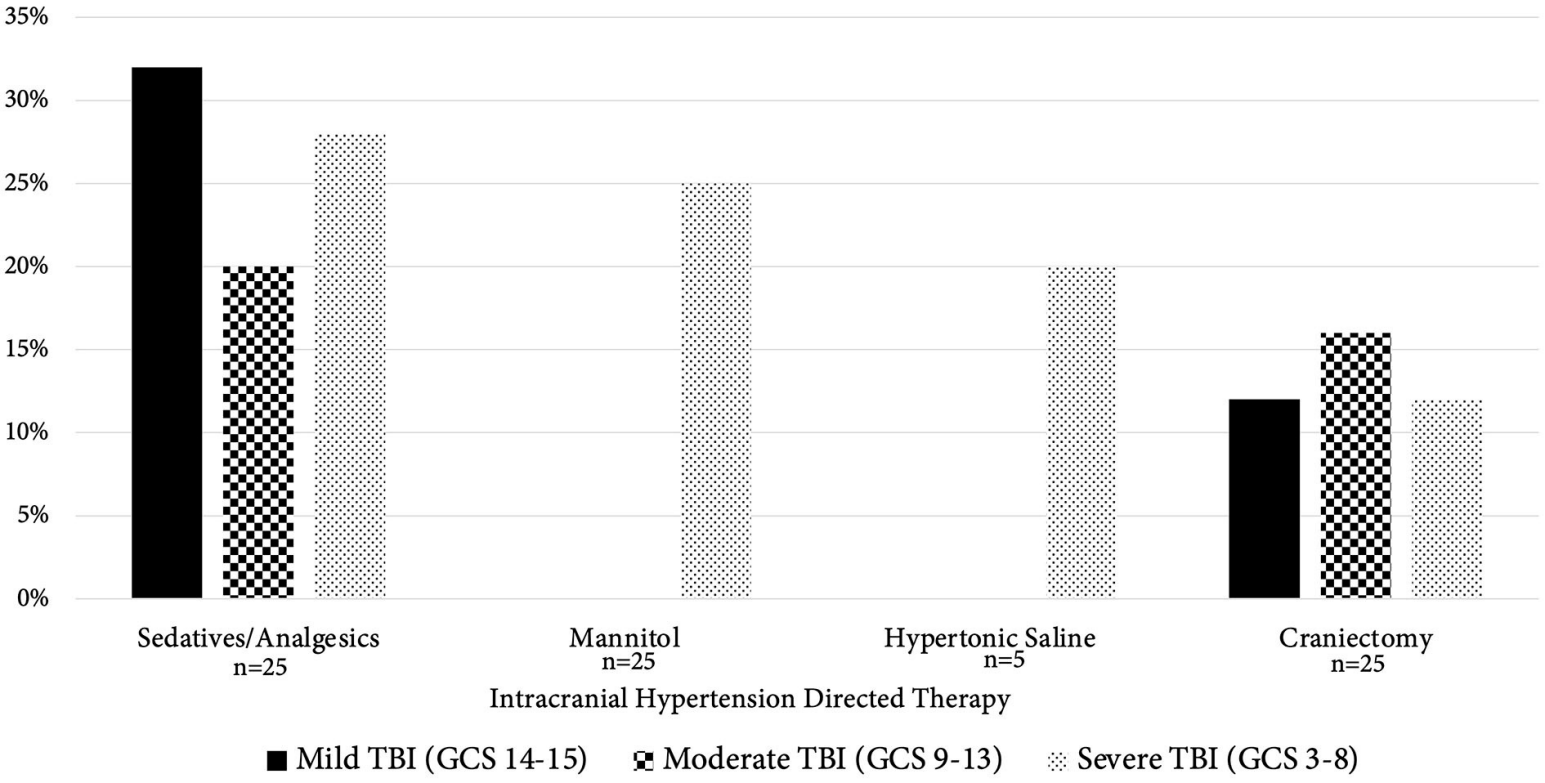
Intracranial hypertension directed therapy compared by TBI severity. This figure compares the use of various intracranial hypertension (ICH) directed therapies between TBI severity groups (mild TBI [GCS score 14–15, black columns], moderate TBI [GCS 9–13, checkerboard columns], severe TBI [GCS 3–8, dotted columns]). The x-axis contains the various therapies separated by TBI severity; the “*n*” under the bar represents total number of patients who had that particular ICH therapy available to them during their hospitalization. Of note, the only therapy not available to all 25 patients who were treated for presumed ICH was hypertonic saline, which was available only for those patients with presumed ICH at the Kenya site (*n* = 5). The y-axis represents the percentage of patients that received ICH directed therapies. Notably, there was no significant difference in utilization of decompressive craniectomy between severity groups. TBI, traumatic brain injury; GCS, Glasgow Coma Scale.

Over half (*n* = 25, 63%) of inpatients were treated for presumed ICH; 64% of those treated (16/25) received multiple therapies during their hospitalization. The most common treatment overall were sedatives/analgesics (21/25, 84%). Mannitol was the hyperosmolar therapy of choice and utilized more frequently than hypertonic saline (7/25 [28%] vs. 1/25 [4%]). Figure 3 outlines individual therapies utilized. Decompressive craniectomy for suspected ICH was performed in 40% (10/25) of patients at a median 1.5 days. There was no significant difference between utilization of decompressive craniectomy between mild vs. moderate vs. severe TBI (*p* = 0.125).

Inpatient physical and occupational therapy had limited availability. Nineteen inpatients had physical therapists available to them, and nine patients (47%) were prescribed PT. Occupational therapists were less available (8/40) and utilized in one patient (Table 3). Of the 10 survivors with a worse PCPC score on discharge, three received PT; none received OT.

### Outcomes and disposition

**Figure 4:**
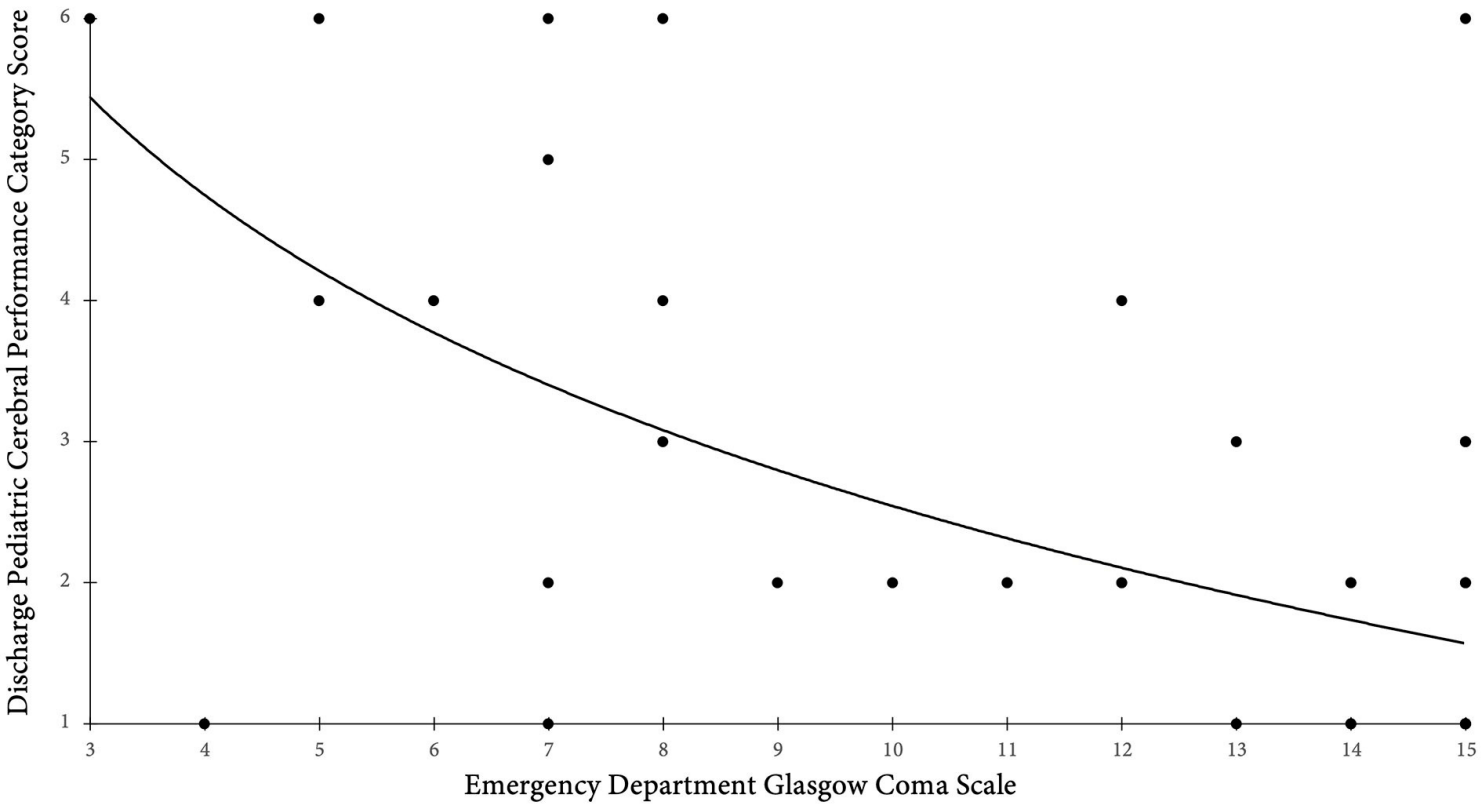
Presenting Glasgow Coma Scale score compared to discharge PCPC score. This figure is a scatter plot with the x-axis containing the presenting Glasgow Coma Scale score and the y-axis representing the PCPC score on discharge. Higher GCS score on arrival to the hospital was associated with a favorable PCPC score, defined as a score of 1–2, on discharge (*p* < 0.001). A line of best fit was abstracted. GCS, Glasgow Coma Scale; PCPC, Pediatric Cerebral Performance Score.

The proportion of children with pre-injury favorable PCPC scores was 95% (38/40); this dropped to 65% (26/40) at hospital discharge (72% in survivors [26/36]) (Table 4). There was no significant difference between proportion of favorable discharge PCPC score between males (17/27) and females (9/13) (*p* = 0.886). Higher GCS score on arrival to the hospital was associated with a favorable PCPC score on discharge (*p* < 0.001) (Figure 4).

**Table 4:** Inpatient outcomes separated by highest level of care.

**Median (IQR) and *n* (%)**	**Ward**	**Operating room**	**PICU**
	***n =* 27**	***n =* 7**	***n =* 6**
Mortality	3 (11)	0 (0)	1 (17)
Discharged home	21 (78)	5 (71)	4 (67)
Discharged to inpatient rehabilitation	3 (11)	2 (29)	1 (16)
**Morbidity**			
Spasticity	4 (17), *n =* 23	1 (14)	2 (50), *n =* 4
Dysautonomia	2 (9), *n =* 23	0 (0)	3 (75), *n =* 4
Hydrocephalus	1 (4), *n =* 23	0 (0)	0 (0), *n =* 4
Nosocomial pneumonia	1 (4), *n =* 23	0 (0)	2 (50), *n =* 4
Nosocomial sepsis	1 (4), *n =* 23	0 (0)	0 (0), *n =* 4
Feeding tube	1 (5), *n =* 22	0 (0)	4 (100), *n =* 4
Tracheostomy	0 (0), *n =* 22	0 (0)	1 (25), *n =* 4

IQR, interquartile range; PICU, pediatric intensive care unit; PCPC, pediatric cerebral performance category.

This table contains detailed outcomes of all admitted patients. Patients are separated in groups determined by the location of their first touchdown clinical space in the hospital. Column header n represents number of patients in each group total, while individual cell n represents number of patients that had that data available. Variable sample sizes are due to missing data.

Hospital mortality was 9% (5/56) overall; four of these deaths occurred in inpatients after hospital admission (Table 4). The patient who died in the ED had polytrauma including severe TBI and presented in shock. Eighty percent (4/5) of patients who died had severe TBI. The fifth death was in a ward patient with moderate TBI who had an intracranial hemorrhage on admission CT. All patients with craniectomy performed survived to discharge, as did 4 of 5 (80%) children who required mechanical ventilation.

## Discussion

This secondary analysis of an observational multicenter study describes clinical practice patterns in pediatric TBI in tertiary care centers in LMICs and reports data that is crucial in laying the foundation for development of guidelines tailored to each region's resources. The main findings of this study include the following: (1) children with TBI were infrequently transported by emergency medical services despite a majority of them requiring transfer to the study site, (2) monitoring and testing tools were not routinely utilized, (3) medical and surgical ICH-directed therapy was frequently used in the absence of ICP monitoring, (4) rehabilitation services are limited, and (5) considerable variability in practice patterns exist that does not appear to be center-specific.

Reports suggest that although most trauma deaths in LMICs occur in the pre-hospital setting, pre-hospital care, when available, remains inefficient and expensive, and thus unaffordable for many (14, 15). Studies from HICs have established the importance of high-quality prehospital care during the “golden hour” of trauma in mitigating secondary brain injury and survival, yet this vital resource frequently remains inaccessible to TBI patients in LMICs (16, 17). Our patients, more than three quarters of whom required interhospital transport, did not have advanced life-support trained personnel available to them during this critical period. This was compounded by the need for interhospital transport over a wide range of distances, a common challenge in LMIC settings (18, 19). These data support the need for investment in expansion of pre-hospital care infrastructure in LMICs.

Evidence from HICs strongly supports maintaining normotension, normoxemia, normothermia, normoglycemia, and normonatremia post-TBI in children to prevent secondary brain injury and negative impact on outcomes (9, 20–22). In our study, a complete set of ED vital signs was obtained in 55% of patients who were admitted. Laboratory values were infrequently monitored in admitted patients. Contributing factors may include lack of evidence-based trauma guidelines and clinical pathways, inadequate staffing, and equipment availability – all postulated reasons that will be explored in future studies. Conversely, head CTs were obtained frequently despite the high proportion of patients with initially normal GCS scores, which may be due to inability to facilitate longer observation periods required when head CT is not obtained. Efforts toward implementing modified adult severe TBI guidelines from HICs that integrate low-cost monitoring and point-of-care testing have led to more efficient care and improved outcomes in single center LMICs, as reported by Kesinger et al. in a trauma center in Neiva, Colombia (23). These data support the importance of standardizing pediatric trauma-focused care in LMICs based on available resources.

Similar to practices in many other LMICs, clinical judgment was used to treat suspected ICH after TBI. The BEST:TRIP (“Benchmark Evidence from South American Trials: Treatment of Intracranial Pressure”) randomized controlled trial, conducted in LMICs prior to PANGEA-DC, reported no difference in outcomes of TBI patients aged 13 years or older with an ICP monitor compared to patients managed using a protocol derived from serial brain CTs and clinical judgement (24). This protocol was recently incorporated into expert consensus-based guidelines for severe TBI in adults (25). In terms of therapies for ICH, hypertonic saline infusion represents a promising therapy for suspected ICH in patients with TBI in LMICs due to its relative affordability, availability of point-of-care testing for blood sodium, and reports of safe peripheral administration and favorable hemodynamic profile compared to mannitol (26–29). Indeed, LMICs have reported increasing availability and safe use of hypertonic saline in children with TBI (30, 31). Of note, in our cohort, mannitol, not hypertonic saline, was the most frequently used hyperosmolar therapy due to lack of availability. Craniectomy was performed in almost half of hospitalized children to treat ICH; this appears to be consistent with practice patterns from LMICs in Asia (32). Data regarding aggregate of medical therapies prior to surgical management were not available in this study. Prospective study is critically needed in LMICs to inform locally-relevant, evidence-based guidelines for invasive and non-invasive assessment and treatment of ICH.

Nearly all patients in our study were previously healthy children, but one in three inpatient survivors had a poor neurological outcome at hospital discharge. Access to rehabilitative services was limited and utilization was infrequent. Literature from HICs suggests early neurorehabilitation improves outcomes in pediatric TBI (33–35). However, it is estimated that 60% of LMICs lack neurorehabilitation services, citing a lack of workforce, training, and equipment needed to provide such services effectively (11). To address this issue, consensus statements suggest conducting workshops for mid-level providers with involvement of patients recovering from TBI and their caregivers, along with utilization of telemedicine and medical partnerships (11).

There are significant limitations to our small study as an exploratory pilot, the first of its kind. These include the small number of centers and patients, asymmetric enrollment numbers (e.g., centers, TBI severity), and short-term outcomes. Data collection was conducted over 4-week periods, a brief period of time that does not address seasonality; although the large cohort of patients enrolled from Ethiopia compared to fewer patients enrolled from the other sites may be due to increased rate of pediatric TBI in the summer months. Details of clinical decision-making or classification are not available. Additionally, we did not collect laboratory or imaging results that were obtained beyond the first 24 h following presentation to the hospital, nor were we able to follow patients as they transitioned between different levels of care. This may have led to, for example, not capturing if patients clinically worsened after initial presentation and GCS score assignment, resulting in initiation of more aggressive ICH-directed therapy. Due to the observational nature of the study, standardization of the definition of clinically determined increased ICP across centers and providers was not possible. Due to small numbers, we are unable to control for variations in patient care strategies and outcome that may have occurred due to injury severity. Finally, as our data represent patient care at more resourced tertiary care centers, findings are less generalizable to district and smaller hospitals and more technologically advanced private hospitals. We may not have captured older pediatric patients cared for in adult hospitals as is more common in LMICs. Further, we may not have captured mild TBI cases that did not require transfer and/or severe cases that resulted in death before transfer.

## Conclusions

The use of clinical monitoring, testing, and treatment of pediatric TBI patients in three LMIC tertiary care referral hospitals were highly variable. Overall mortality in our study was 9% and a quarter of survivors had an unfavorable PCPC score on discharge, which is consistent with epidemiological reports of high morbidity and mortality from TBI in Kenya, Ethiopia, and Rwanda (36–38). This mortality and morbidity rate is triple and double, respectively, what is reported in children in the United States, highlighting continued disparities between HICs and LMICs (39, 40). This exploratory study supports the need for multi-center prospective studies in LMICs to support evidence-based guidelines for the care of pediatric TBI patients of various severities across the care continuum.

## Data availability statement

The raw data supporting the conclusions of this article will be made available by the authors, without undue reservation.

## Ethics statement

The studies involving human participants were reviewed and approved by IRB at the University of Pittsburgh Individual Ethics Committees at each site. Written informed consent to participate in this study was provided by the participants' legal guardian/next of kin.

## Author contributions

MR made substantial contributions to the conception and design of the work, the analysis and interpretation of the data for the work, drafted the work and revised it critically for important intellectual content, approved the final version to be published, and agrees to be held accountable for all aspects of the work in ensuring that questions related to the accuracy or integrity of any part of the work are appropriately investigated and resolved. SH, AS, TL, EO, RK, PW, AA, LT, and TB made substantial contributions to the conception and design of the work, the acquisition of the data for the work, revised it critically for important intellectual content, approved the final version to be published, and agree to be held accountable for all aspects of the work in ensuring that questions related to the accuracy or integrity of any part of the work are appropriately investigated and resolved. AF, KR, CW, RT, DA, PK, and EF made substantial contributions to the conception and design of the work, the analysis and interpretation of the data for the work, revised it critically for important intellectual content, approved the final version to be published, and agree to be held accountable for all aspects of the work in ensuring that questions related to the accuracy or integrity of any part of the work are appropriately investigated and resolved. All authors contributed to the article and approved the submitted version.

## Funding

The parent study was funded by the Laerdal Foundation.

## Conflict of interest

The authors declare that the research was conducted in the absence of any commercial or financial relationships that could be construed as a potential conflict of interest.

## Publisher's note

All claims expressed in this article are solely those of the authors and do not necessarily represent those of their affiliated organizations, or those of the publisher, the editors and the reviewers. Any product that may be evaluated in this article, or claim that may be made by its manufacturer, is not guaranteed or endorsed by the publisher.

## References

[1] Clark H Coll-Seck A Banerjee A Peterson S Dalglish S Ameratunga S. A future for the world's children? A WHO–UNICEF–lancet commission. The Lancet. (2020) 395:605–58. 10.1016/S0140-6736(19)32540-1 32085821

[2] United Nations Children's Fund. World Health Organization; World Bank Group. United Nations Population Division. Levels & Trends in Child Mortality: Report 2019. New York, NY: United Nations Inter-Agency Group for Child Mortality Estimation (2019).

[3] Roth G Abate D Abate K Abay S Abbafati C Abbasi N. Global, regional, and national age-sex-specific mortality for 282 causes of death in 195 countries and territories, 1980–2017: a systematic analysis for the global burden of disease study 2017. Lancet. (2018) 392:1736–88. 10.1016/S0140-6736(18)32203-7 30496103 PMC6227606

[4] Dewan M Rattani A Gupta S Baticulon R Hung Y Punchak M. Estimating the global incidence of traumatic brain injury. J Neurosurg. (2019) 130:1080–97. 10.3171/2017.10.JNS17352 29701556

[5] Landes M Venugopal R Berman S Heffernan S Maskalyk J Azazh A. Epidemiology, clinical characteristics, and outcomes of head injured patients in an Ethiopian emergency centre. Af J Emerg Med. (2017) 7:130–4. 10.1016/j.afjem.2017.04.001 30456124 PMC6234141

[6] Emejulu JKC Isiguzo CM Agbasoga CE. Traumatic brain injury in the accident and emergency department of a tertiary hospital in Nigeria. East Central Af J Surg. (2010) 15:28–38.

[7] Dewan M Mummareddy N Wellons J Bonfield C. Epidemiology of global pediatric traumatic brain injury: qualitative review. World Neurosurg. (2016) 91:497–509. 10.1016/j.wneu.2016.03.045 27018009

[8] De Silva M Roberts I Perel P Edwards P Kenward M Fernandes J. Patient outcome after traumatic brain injury in high-, middle- and low-income countries: analysis of data on 8927 patients in 46 countries. Int J Epidemiol. (2008) 38:452–8. 10.1093/ije/dyn189 18782898

[9] Kochanek P Tasker R Carney N Totten A Adelson P Selden N. Guidelines for the management of pediatric severe traumatic brain injury, third edition. Pediat Crit Care Med. (2019) 20:280–9. 10.1097/PCC.0000000000001736 30830016

[10] Halstead M Walter K Moffatt K LaBella C Brooks M Canty G. Sport-related concussion in children and adolescents. Pediatrics. (2018) 142:3074. 10.1542/peds.2018-3074 30420472

[11] Corley J Barthélemy E Lepard J Alves J Ashby J Khan T. Comprehensive policy recommendations for head and spine injury care in low- and middle-income countries. World Neurosurg. (2019) 132:434–6. 10.1016/j.wneu.2019.08.240 31810143

[12] Fink E von Saint Andre-von Arnim A Kumar R Wilson P Bacha T Aklilu A. Traumatic brain injury and infectious encephalopathy in children from four resource-limited settings in Africa. Pediat Crit Care Med. (2018) 19:649–57. 10.1097/PCC.0000000000001554 29664874

[13] Fiser D. Assessing the outcome of pediatric intensive care. J Pediatr. (1992) 121:68–74. 10.1016/S0022-3476(05)82544-2 1625096

[14] Mock C Jurkovich G Nii-Amon-Kotei D Arreola-Risa C Maier R. Trauma mortality patterns in three nations at different economic levels. J Trauma: Inj Infect Crit Care. (1998) 44:804–14. 10.1097/00005373-199805000-00011 9603081

[15] Kiragu A Dunlop S Mwarumba N Gidado S Adesina A Mwachiro M. Pediatric trauma care in low resource settings: challenges, opportunities, and solutions. Front Pediat. (2018) 6:155. 10.3389/fped.2018.00155 29915778 PMC5994692

[16] Suryanto Plummer V Boyle M. EMS Systems in lower-middle income countries: a literature review. Prehos Disast Med. (2016) 32:64–70. 10.1017/S1049023X1600114X 27938449

[17] Chesnut RM Marshall LF Klauber MR Blunt BA Baldwin N Eisenberg HM. The role of secondary brain injury in determining outcome from severe head injury. J Trauma. (1993) 34:216–22. 10.1097/00005373-199302000-00006 8459458

[18] Hodkinson P Argent A Wallis L Reid S Perera R Harrison S. Pathways to care for critically ill or injured children: a cohort study from first presentation to healthcare services through to admission to intensive care or death. PLoS ONE. (2015) 11:e0145473. 10.1371/journal.pone.0145473 26731245 PMC4712128

[19] Callese TE Richards CT Shaw P Schuetz SJ Paladino L Issa N. Trauma system development in low- and middle-income countries: a review. J Surg Res. (2014) 193:300–7. 10.1016/j.jss.2014.09.040 25450600

[20] Vavilala MS Bowen A Lam AM Uffman JC Powell J Winn HR Rivara FP. Blood pressure and outcome after severe pediatric traumatic brain injury. J Trauma. (2003) 55:1039–44. 10.1097/01.TA.0000101759.23607.57 14676648

[21] Kannan N Wang J Mink RB Wainwright MS Groner JI Bell MJ. Timely hemodynamic resuscitation and outcomes in severe pediatric traumatic brain injury: preliminary findings. Pediat Emerg Care. (2018) 34:325–9. 10.1097/PEC.0000000000000803 27387972 PMC5233691

[22] Smith M Baltazar GA Pate A Akella K Chendrasekhar A. Hyponatremia on initial presentation correlates with suboptimal outcomes after traumatic brain injury. Am J Surg. (2017) 83:e126–8. 10.1177/000313481708300408 28424116

[23] Kesinger MR Nagy LR Sequeria DJ Charry JD Puyana JC Rubiano AM. standardized trauma care protocol decreased in-hospital mortality of patients with severe traumatic brain injury at a teaching hospital in a middle-income country. Injury. (2014) 45:1350–4. 10.1016/j.injury.2014.04.037 24861416

[24] Chesnut RM Temkin N Carney N Dikmen S Rondina C Videtta WP. A trial of intracranial-pressure monitoring in traumatic brain injury. N Eng J Med. (2012) 367:2471–81. 10.1056/NEJMoa1207363 23234472 PMC3565432

[25] Chesnut RM Temkin N Videtta W Petroni G Lujan S Pridgeon J. Consensus-based management protocol (CREVICE Protocol) for the treatment of severe traumatic brain injury based on imaging and clinical examination for use when intracranial pressure monitoring is not employed. J Neurotrauma. (2020) 37:1291–9. 10.1089/neu.2017.5599 32013721 PMC7249475

[26] Shein SL Ferguson NM Kochanek PM Bayir H Clark RSB Fink E. Effectiveness of pharmacological therapies for intracranial hypertension in children with severe traumatic brain injury – results from an automated data collection system time-synched to drug administration. Pediat Crit Care Med. (2016) 17:236–45. 10.1097/PCC.0000000000000610 26673840 PMC4779724

[27] Brenkert TE Estrada CE McMorrow SP Abramo TJ. Intravenous hypertonic saline use in the pediatric emergency department. Pediatr Emerg Care. (2013) 29:71–3. 10.1097/PEC.0b013e31827b54c3 23283268

[28] Luu JL Wendtland CL Gross MF Mirza F Zouros A Zimmerman GJ. Three percent saline administration during pediatric critical care transport. Pediatr Emerg Care. (2011) 27:1113–7. 10.1097/PEC.0b013e31823aff59 22134236

[29] Perez C.A. Figueroa S.A. Complication rates of 3% hypertonic saline infusion through peripheral intravenous access. J Neurosci Nurs. (2017) 49:191–5. 10.1097/JNN.0000000000000286 28471928

[30] Wooldridge G Hansmann A Aziz O O'Brien N. Survey of resources available to implement severe pediatric traumatic brain injury management guidelines in low- and middle-income countries. Child's Nervous System. (2020) 36:2647–55. 10.1007/s00381-020-04603-9 32300872

[31] Mohammad N Banu S Brown N Kaleem S Akhtar S ul-Haq A. Hypertonic saline: safe therapy for children with acute brain insult in emergency department of low and middle income country. J Pediatric Care. (2017) 3:1–5. 10.21767/2471-805X.100024

[32] Chong SL Dang HX Ming MX Mahmood M Zheng CQS Gan CS. traumatic brain injury outcomes in 10 Asian pediatric intensive care units: a pediatric acute and critical care medicine Asian network (PACCMAN) retrospective study. Pediat Crit Care Med. (2021) 22:401–11. 10.1097/PCC.0000000000002575 33027240

[33] Tepas JJ Leaphart CL Pieper P Beaulieu CL Spierre LR Tuten JD. The effect of delay in rehabilitation on outcome of severe traumatic brain injury. J Pediatr Surg. (2008) 44:368–72. 10.1016/j.jpedsurg.2008.10.089 19231536

[34] Eilander HJ Wijnen VJM Scheirs JGM de Kort PLM Prevo AJH. Children and young adults in a prolonged unconscious state due to severe brain injury: Outcome after an early intensive neurorehabilitation programme. Brain Injury. (2005) 19:425–36. 10.1080/02699050400025299 16101265

[35] León-Carrión J Machuca-Murga F Solís-Marcos I León-Domínguez U Domínguez-Morales M. The sooner patients begin neurorehabilitation, the better their functional outcome. Brain Inj. (2013) 27:1119–23. 10.3109/02699052.2013.804204 23895589

[36] Kinyanjui B. Traumatic Brain Injury in Kenya. SAGE Open. (2016) 6:215824401663839. 10.1177/2158244016638392

[37] Krebs E Gerardo C Park L Nickenig Vissoci J Byiringiro J Byiringiro F. Mortality-associated characteristics of patients with traumatic brain injury at the university teaching hospital of Kigali, Rwanda. World Neurosurg. (2017) 102:571–82. 10.1016/j.wneu.2017.03.001 28336445 PMC5681277

[38] Assele D Lendado T Awato M Workie S Faltamo W. Incidence and predictors of mortality among patients with head injury admitted to Hawassa university comprehensive specialized Hospital, Southern Ethiopia: a retrospective follow-up study. PLOS ONE. (2021) 16:e0254245. 10.1371/journal.pone.0254245 34411116 PMC8376017

[39] Pelletier JH Rakkar J Simon D Au AK Fuhrman DY Clark RSB. Association between pediatric TBI mortality and median family income in the United States: a retrospective cohort study. Lancet Reg Health - Ams. (2022) 5:100164. 10.1016/j.lana.2021.100164 35252952 PMC8896657

[40] Berger RP Beers SR Richichi R Wiesman D Adelson PD. serum biomarker concentrations and outcome after pediatric traumatic brain injury. J Neurotrauma. (2007) 24:1793–801. 10.1089/neu.2007.0316 18159990

