# Supplementary material for: An exploratory assessment of the management of pediatric traumatic brain injury in three centers in Africa
Source: Front Pediatr. 2022 Aug 17;10:936150. doi: 10.3389/fped.2022.936150 (PMC9428450; doi:10.3389/fped.2022.936150)
Supplement: Supplementary file 1 [file Table_1.docx]

**Supplementary Material**

Electronic Supplementary Material Table 1. Center Characteristics

|  | Ethiopia  N=38 | Kenya  N=8 | Rwanda  N=12 |  |
| --- | --- | --- | --- | --- |
| City | Addis Ababa | Nairobi | Kigali |  |
| No. pediatric beds | 176 | 450 | 57 |  |
| No. pediatric ED admissions/year | 3,000 | 600-800 | 2,400 |  |
| No. pediatric hospital admissions/year | 7,885 | 4,250 | 1,230 |  |
| No. pediatric ICU beds | 4 | 10 | 5 |  |
| No. pediatric ICU admissions/year | 270 | 300 | 108 |  |
| ICU nurse to patient ratio | 1:1^*^ | 1:2 | 1:1 |  |
| All centers have supplemental oxygen, IV fluids, mechanical ventilation, cardiac monitoring, X-ray, CT scanner, and Neurosurgery. General wards had higher patient to staff ratios and typically did not have capacity for continuous monitoring for all patients. | | | | |
| No center has intracranial pressure monitoring or a trauma response team | | | | |

Legend: *=represents nursing ratio with a mechanically ventilated patient. No. = number of, ED = emergency department, ICU = intensive care unit, IV = intravenous, CT = computed tomography.

Caption: This table describes characteristics of the three centers in our study: Tikur Anbessa Hospital of Addis Ababa University in Addis Ababa, Ethiopia, Kenyatta National Hospital in Nairobi, Kenya, and University Teaching Hospital in Kigali, Rwanda.
